# Pilot Study About the Importance of the Active Role of Roma Students: Improving the Health of Bulgarian Children from the Roma Minority Group Through Nutrition and Probiotics

**DOI:** 10.3390/healthcare13111314

**Published:** 2025-06-01

**Authors:** Bozhidarka Radoslavova Hadzhieva, Marin Kostadinov Baltov, Daniela Ivova Taneva, Atanas Denev Luizov, Milen Ventsislavov Dimitrov, Valentina Boyanova Petkova-Dimitrova

**Affiliations:** 1 Medical College, Medical University of Plovdiv, 15a Vasil Aprilov Blvd., 4000 Plovdiv, Bulgaria; 2 Department of Forensic Medicine and Deontology, Faculty of Medicine, Medical University of Plovdiv, 15a Vasil Aprilov Blvd., 4000 Plovdiv, Bulgaria; marin.baltov@mu-plovdiv.bg; 3 Department of Nursing Care, Faculty of Public Health, Medical University of Plovdiv, 15a Vasil Aprilov Blvd., 4000 Plovdiv, Bulgaria; daniela.taneva@mu-plovdiv.bg; 4 Department Marketing, Faculty of Business Studies, Burgas Free University, 62 San Stefano Street, 8001 Burgas, Bulgaria; luizov@bfu.bg; 5 Department Pharmaceutical Technology and Biopharmacy, Medical University of Sofia, 2 Dunav St., 1000 Sofia, Bulgaria; mdimitrov@pharmfac.mu-sofia.bg; 6 Department of Social Pharmacy, Faculty of Pharmacy, Medical University of Sofia, 2 Dunav St., 1000 Sofia, Bulgaria; petkovav1972@yahoo.com

**Keywords:** healthcare, students, education, probiotics, children, Roma

## Abstract

**Background/Objective:** The priority task of each country is to ensure the protection of and improvement in its people’s health. One of the key aspects of health is related to food culture, consuming foods that ensure growth and normal development in a person and also prevent diseases. The consumption of foods that contain essential nutrients and functional foods, which include those containing probiotics, is the basis of a healthy diet. **Methods:** A structured anonymous interview was conducted with 90 parents from the Roma minority group to assess the nutritional culture of children from these families. The knowledge of Roma parents about probiotics, as well as their attitudes to apply them to their children, was examined. The survey was based on a questionnaire that was distributed among the respondents, Roma students, under the guidance of a mentor. Roma students study health specialties and are also participants in a Scholarship Programme. **Results:** We have established that the daily diet of children from this minority group consists of foods containing hydrogenated fats and sugar, which was indicated by 53.3% of respondents, and only 28.9% of respondents noted that their children consume fresh fruit every day. We have established that less than half of the children (35.6%) consume yoghurt daily as a source of valuable probiotics. We applied a nonparametric Kruskal–Wallis test and found statistically significant differences in the respondents’ knowledge of probiotics (χ^2^ = 16.186, *p* = 0.001): those receiving secondary specialized education were better informed, but education has not affected their knowledge of the health benefits of probiotics (χ^2^ = 5.462, *p* = 0.141). Children from minority groups tend to eat unhealthy foods. **Conclusions:** The role of Roma students studying health specialties as participants in the Scholarship Programme is to assist parents in forming a nutritional culture in their children. Roma students, due to their ethnic and cultural proximity and potential to be health professionals, contribute to sustainable health improvements among the Roma community as a whole.

## 1. Introduction

The Roma people settled in Bulgaria at different times, coming from various places around the world. Owing to this, the Roma community in Bulgaria is characterized by significant ethnical, language, and religious diversity [1]. These differences also affect the health of the Roma people [2]. The factors that have an effect on the health condition of people from minority groups include the following: poverty, a low level of education, poor living and sanitary conditions, and malnutrition. Poverty is the main factor. We could define this poverty as being hereditary. It also becomes worse as a result of mass and continuous unemployment. Poverty has a direct effect on the health of the Roma people. Due to a lack of money, people from minority groups often cannot purchase prescribed medicines, or they purchase them at a later stage, and therefore the treatment is not sufficiently effective. In general, hospital treatment is very rare among the Roma community owing to the inability of Roma people to pay the established hospital fees. Self-treatment is the most common form of treatment among the residents of Roma neighbourhoods [3].

The health of Roma people is significantly poor compared to the health of most of the European population [4].

Despite incomplete and scarce data about the health status of the Roma population, it is known that life expectancy, children’s death rate, and the morbidity indices among children and adults are worse for Roma people compared to the majority of the population [5]. In a study conducted in Bulgaria, Hungary, and Romania, it has been documented that Roma people are much more inclined to report a deteriorated health condition under any of the indices compared to non-Roma people [6].

Nutrition habits and behaviour are formed at a very young age, which necessitates taking reasonable measures to ensure the timely acquisition of proper nutrition habits in order to prevent any health problems and future diseases [7,8]. The formation of proper nutritional behaviour from a child’s early age is an important factor in their overall development [9].

Children from poor families are more likely to consume food which does not contain a sufficient quantity of nutrients and, as a result, they suffer from various diseases (for example, overweight and obesity) owing to the unhealthy nutrition habits that they have acquired [10]. We also need to consider the fact that improper nutrition is related to serious health consequences, where the treatment of which may involve vast expenses incurred by health systems worldwide [11]. A number of studies conducted in European countries indicate that children’s eating habits are not ideal and are difficult to change, as they have been formed at an early age, and the role of health professionals in this respect is essential [12,13]. Even if children eat well, the intestinal microbiome plays a key role in human health. A number of studies highlight the relationship between the microbiota and the pathophysiology of various diseases. Taking probiotics can help maintain health by stabilizing the intestinal microbiota [14]. In 1953, Werner Kollath, a German scientist, introduced the term “probiotic”, which means “active substances that are essential for the healthy development of life” [15]. Over the years, the definition of probiotics underwent a number of changes, until the Food and Agriculture Organization (FAO) of the United Nations supported by the World Health Organization (WHO) convened an expert group that defined probiotics as “live microorganisms which, when administered in adequate amounts, confer a health benefit on the host” [16].

Another important factor affecting the health of Roma people is low level of education. In Bulgaria, there are substantial differences between Roma people and the majority of the population with regard to the levels of enrolment in any form of education [1]. As a result, the educational policy in Bulgaria is aimed at improving the education of the Roma people. A number of projects supported by the Open Society Institute and the Roma Educational Fund (REF) have been dedicated to school segregation, supporting Roma children in their integration in desegregated schools outside their neighbourhoods [17].

The main priority of the Roma Educational Fund is to support the development and implementation of educational policies that contribute to the inclusion of Roma people in the educational system. The Roma Educational Fund also supports a project aimed at improving the access of Roma people to universities and increasing their level of participation in scientific research [17].

Within the scope of the Active Citizens Fund Bulgaria, there is a functioning component for providing scholarships to young people of Roma origin who study health majors (Scholarship Programme). The Scholarship Programme is managed by the Social Alternative Trust Foundation. The Scholarship Programme is intended to last six years [18]. Various activities are held within the scope of the Scholarship Programme. For example, an activity of this type is the organization of a small community initiative. The small community initiatives are organized on the grounds of ideas proposed by the scholarship students included in the Programme, with the support of their mentors. They aim to resolve certain community issues identified by the participants—both scholarship students and mentors. Small community initiatives are being implemented in different regions of the country to prevent diseases and promote healthy lifestyles. For example, a similar initiative was conducted among minority parents to build healthy eating habits and culture in children. In addition, Roma students also inform the parents about the types of probiotics and their effect on the body; the foods that contain probiotics; and if additional intake is needed, how they could obtain them in a concentrated form such as drops, powders, chewable tablets, etc.

By organizing information meetings/providing brochures, the Roma students, under the guidance of a mentor, actively participate in health prevention programmes for adolescents and minority groups as a whole.

The aim of this study is to present the role of Roma students in conducting community initiatives, where they present topics about healthy lifestyles with an emphasis on the health of minority children. We also aim to assess the nutritional culture and habits of children from Roma families in Bulgaria, as well as the parents’ knowledge about the benefits of probiotics for the microbiota and overall health in humans.

## 2. Materials and Methods

### 2.1. Study Design and Setting

Structured interviews were conducted among parents of children from the Roma community. The study population includes parents within the Roma community who have children and adolescents up to 18 years of age. Three settlements in the vicinity of the second largest city in Bulgaria—Plovdiv—were covered: Saedinenie, Perushtits, and Parvomay.

Based on data provided by the Civil Registration and Administrative Servicing Department, the number of people residing permanently in these towns in 2023 is as follows: town of Saedinenie—5580 people; town of Perushtitsa—4992 people; and town of Parvomay—13,010 people.

This study was conducted by students participating in the Scholarship Programme managed by the Social Alternative Trust Foundation under the guidance of a mentor. Scholarship students have shown great achievements during their education. As participants in the Programme, they have taken public engagement to spread awareness and teach healthy lifestyles among this minority population.

The data were collected within the period March–May 2023. Exclusion criteria include the following: illiteracy; juveniles.

### 2.2. Survey Instrument

The research instrument is a questionnaire that includes statements about the diet of the children from Roma families. The questionnaire (Supplementary Material S1) includes five sections and one subsection as follows: demographic characteristics of the Roma respondents (gender, age, education, place of residence, number of children in the family, age group of the children); presence/absence of chronic diseases in children; nutritional diet; eating style; and knowledge and attitude of parents to give probiotics to their children. The questions in the third section focus on the intake of different types of food per week, with a subsection related to specifying the type of fat most often used when preparing meals. In the fourth section, the questions are related to the way of eating (at home, in a public establishment, on the go, etc.) of the children from Roma families. The questions in the fifth section concern the knowledge of parents about probiotics and their use by children.

The questionnaire contains information about the purpose of this study and a note stating that by completing it, the parents give their informed consent for their data to be processed and used for scientific purposes.

### 2.3. Statistical Method

The respondents were described in terms of absolute and relative frequencies, means, and standard deviations.

In this paper, the nonparametric Kruskal–Wallis H test is used. This rank-based test is employed to compare more than two independent samples to determine if they originate from the same distribution. The Kruskal–Wallis test serves as a robust alternative to the one-way analysis of variance (ANOVA), particularly when the assumptions of normality required by ANOVA cannot be satisfied. Unlike parametric tests, the Kruskal–Wallis test does not assume normality of the data; however, it does require that the samples be independent of each other.

The Kruskal–Wallis test operates by ranking all the data from all the groups together and then evaluating whether the sum of the ranks differs significantly across the groups. If the Kruskal–Wallis statistic indicates a significant result, it suggests that at least one of the samples originates from a different distribution. In such cases, subsequent nonparametric multiple comparison tests can be conducted to pinpoint the specific groups that differ from one another. The strength of the Kruskal–Wallis test lies in its flexibility and broad applicability across various fields of research, making it an essential tool for nonparametric statistical analysis.

Statistical analysis was performed with IBM SPSS 27.0 (IBM Corp., Armonk, NY, USA). We have applied the nonparametric Kruskal–Wallis test to four hypotheses to analyse the relationship between the parents’ knowledge about probiotics and the demographic characteristics—age, education, and the number of children.

The following subsections present the hypotheses guiding this research, detailing the relationships between the variables in this study. We define four hypotheses (H1, H2, H3, H4) as follows in Table 1.

## 3. Results

The research sample consists of 90 respondents of Roma origin, where 81.1% are women and 18.9% are men. The participants’ ages range from 19 to 57. The mean age of the female respondents is 31.07 years (SD = 5.287), while that of the male respondents is 36.71 years (SD = 7.655). Half of the respondents have two children, 43.3% have one child, and a small proportion (6.7%) report having three children. The demographic characteristics of the surveyed Roma parents are presented in Table 2.

Most of the respondents have completed primary education (63.2%; *n* = 55) and secondary education (19.5%; *n* = 17); 13.8%; *n* = 12 of the parents have a specialized secondary education and only 3.4%; *n* = 3 have a Bachelor’s degree. There are respondents who are literate but did not indicate what level of education they have. Half of the surveyed parents reported that they have two children in their family (50%; *n* = 45), 43.3%; *n* = 39 answered that they have one child, and families with three children constitute 6.7%; *n* = 6.

Most of the children of the parents interviewed are currently in elementary and/or primary school (Table 3).

When asked “Does your child often get colds?”, more than half of the studied people (56.7%; *n* = 53) stated that their children are often ill with colds, and 43.3% *n* = 39 answered “no”. We could assume that the reason for the more frequent cases of cold is related to the age of the children, most of whom are in elementary and primary school.

The respondents were asked whether their children suffer from any of the following chronic diseases: diseases of the stomach and oesophagus (gastritis/ulcer); obesity, as well as elevated blood sugar and elevated blood pressure (metabolic syndrome); diabetes; high blood pressure (hypertension); or heart disease. The names of the diseases written above have been formulated in this way considering that the respondents are not medical specialists. We should also clarify that the clinical history of the subjects (children) has not been reviewed since it is not the subject of this study. The question related to chronic diseases was included in the survey as one of the reasons for their occurrence is related to the human diet. The existence of chronic diseases among the children of the respondents is presented in Table 4.

The percentage of respondents who answered the question about chronic diseases was 20%, *n* = 18. We could assume that not every parent of minority children surveyed answered this question. We have not reviewed the clinical history of the respondents’ children, as this is not the subject of our study (as mentioned above). One of the causes of the diseases listed in the table is poor nutritional culture and habits. These diseases also require adherence to a nutritious diet [19,20]. Parents, together with health professionals, should make efforts to build healthy eating habits from early childhood [21]. The section related to diet includes types of foods consumed by children from Roma families, specifying how many times a week they eat them (Table 5).

The respondents answered questions about the types of food included in their children’s diet (Table 5).

The majority of children (40%) of the respondents consume non-perishable products (sausages, pâté, etc.) daily. The percentage of the children (53.3%) who consume products containing hydrogenated fats and sugar (wafers, biscuits, cakes) daily is high. A total of 46.7% of the children consume products such as pasta/spaghetti more than twice a week. The percentage of children (50%) who eat milk and dairy products more than twice a week is predominant. In total, 55.6% of the children have meat products on their table more than twice a week. A positive aspect of the children’s diet is that 51.7% of them consume fish once a week. A total of 38.9% of the children eat fresh fruits (e.g., apples, oranges, blueberries, etc.) every day and 31.1% eat vegetables (e.g., peppers, carrots, spinach, etc.). Children who never eat fresh fruits and vegetables constitute 5.6%, as is the percentage of children who only consume products containing carbohydrates and “hidden” sugars once a week.

Table 6 presents the findings on respondents’ knowledge, beliefs, and practices regarding probiotics. The data reveal that only 34.4% of the participants reported familiarity with probiotics, while a substantial proportion (45.6%) were unsure, and 20% had no awareness of them. Regarding beliefs about the health benefits of probiotics, the majority (56.7%) expressed uncertainty; 34.4% believed they were beneficial; and 8.9% did not perceive any health benefits. Concerning the use of probiotics for their children, 34.4% of respondents indicated that their child had consumed probiotics, 29.4% were uncertain, and 12.2% reported that their child had not taken them.

The analysis of the relationship between parents’ knowledge of probiotics and the demographic characteristics—age, education, and number of children—is presented through the nine hypotheses, for which the Kruskal–Wallis test has been applied (Table 7).

H1: The results of the test have shown that there are statistically significant differences in the knowledge between the three age groups. They have the highest knowledge in the lowest age group (up to 29 years), while in the next two the knowledge decreases, and in the oldest (over 39 years), the most frequent answer is “I don’t know what probiotics are”. H2: The results of the test have shown that there are statistically significant differences in the assessment of the usefulness of probiotics between the three age groups. Parents in the first age group have rated probiotics as useful (definitely or to some extent), while those in the other two groups had a rather neutral or negative attitude. H3: The results of the test have shown that there are statistically significant differences in the knowledge between educational groups. Respondents with higher than primary education have the highest knowledge—these are mostly parents with secondary-specialized education. H4: The results of the test have shown that families with one child have statistically significant differences in the knowledge compared to those with two or more children. The result of the hypothesis test shows that in the group of parents with one child, the median is “we know what the probiotics are like”, while in families with two or more children, the median is “unsure”.

The analysis of the hypotheses shows that respondents in the lowest age group (up to 29 years) have better knowledge about what probiotics are and their usefulness for human health. The age of the Roma parents, as well as the number of children in the family, does not affect their attitude to the use of these drugs among their children. Respondents with a higher educational level have better knowledge about probiotics. Surprisingly, despite their low level of education, Roma parents give probiotics to their children, which we could explain with their trust in health professionals, including Roma students.

## 4. Discussion

This pilot study presents an analysis conducted among Roma parents regarding their knowledge about probiotics and also examines the eating habits of their children. There is a need to improve the eating culture among children from minority groups. Roma parents need to gain more knowledge about the beneficial effects of probiotics, foods containing probiotics, as well as the possibility of taking them in concentrated form such as powders, capsules, or tablets.

Healthy eating among adolescents is part of health education, health culture, and health literacy. A number of studies have demonstrated the mechanisms through which education influences health. Empirical studies have established correlations between education and healthy behaviour—for example, better educated people smoke less, exercise, eat healthily, etc. [22,23].

In our study, only 3.4% of the respondents had undergone higher education, and most of the participants had received primary education (63.2%). Low education and professional qualifications are some of the main factors for poor health culture among the Roma population in Bulgaria [1]. We could assume that the low level of education of parents is one of the barriers to modelling healthy eating habits in children from minority groups. The formation of healthy eating habits should start from an early age and be a model of life in the family. The food preferences and eating behaviour of parents play a role in the eating pattern of children [24].

Based on the conducted study, we established that most of the parents interviewed have not formed healthy and balanced nutrition habits as part of the nutrition culture of their families. It has been documented that in Bulgaria and other countries as well, the Roma ethnic group has poor eating habits and low standards of personal and public hygiene, which are prerequisites for an unhealthy lifestyle in general [25]. Our study has showed that more than half of the parents (53.3%) stated that their children consume foods containing hydrogenated fats and sugar daily. Also, 46.7% of the respondents indicated that more than twice a week their children’s diet includes pasta. Children from minority groups consume foods that are sources of so-called “empty calories” daily. Regular consumption of these foods is associated with adverse effects such as increased blood pressure, insulin resistance and diabetes mellitus, obesity, etc. [26].

Food products containing hydrogenated fats and sugar are a daily component of the diet of children, while less than ⅓ of respondents indicating that their children consume fresh fruits and vegetables every day. The influence of parents in the early childhood stage of life is essential, which is an extremely good opportunity to encourage children to adopt a healthy lifestyle. It is very important that parents adhere to a healthy lifestyle and diet. For example, a mother who likes to drink cola will find it difficult to keep her own child away from it [27].

Regarding the consumption of milk and dairy products, we reported a relatively low intake—35.6% of respondents stated that such food products are present in their children’s daily diet. Increasing evidence confirms that the consumption of yoghurt has a beneficial effect on the healthy growth and development of children [28].

Yoghurt has long been recognized as a carrier food for probiotic microorganisms. By consuming yoghurt, consumers can ingest large amounts of probiotic cells for a therapeutic effect [29,30]. A number of studies confirm that the frequent consumption of yoghurt lowers the risk factors for cardiovascular diseases, reduces the risk of diabetes, improves the development of the host’s immunity, and also reduces the risk of dysbiosis [31].

Probiotics are extensively utilized in the production and enhancement of fermented dairy products. Additionally, freeze-dried bacteria are offered on the pharmaceutical market in the form of tablets, capsules, and powders [32]

Other foods dominated by probiotics are kefir (a fermented milk product), sauerkraut, sourdough bread, olives, and pickles [33]. A number of studies have described the beneficial effects of probiotics such as improving the health of the intestines, enhancing the immune response, reducing serum cholesterol, and even preventing cancer [34,35,36].

A research study carried out in Finland indicates that the use of probiotics may be an effective approach to reduce the incidence of paediatric patients hospitalized due to rotavirus diarrhoea [37]. Probiotics are effective as adjuncts to the prescribed therapy in the treatment of gastrointestinal disorders such as necrotizing enterocolitis (NEC), antibiotic-associated diarrhoea, recurrent Clostridium difficile colitis, Helicobacter pylori infections, and inflammatory bowel disease (IBD) [38]. A clinical study has shown the efficacy of Lactobacillus paracasei GY-1 in reducing the intestinal toxicity of Colchicine, which is used to treat gout attacks [39]. Although there is a lot of evidence in the literature about the ability of probiotics to modify the host immune response, affect gastrointestinal infections, aid irritable bowel syndrome, help lactose intolerance, etc., clinical studies are still needed to further improve our understanding of how probiotics work [40].

In this context, we studied the knowledge of Roma parents about probiotics and their benefits for the microbiota and human health. Using the Kruskal–Wallis test, we tested the four hypotheses. When testing the hypotheses regarding education and knowledge about probiotics, we found that Roma parents with a higher level of education are the most knowledgeable about probiotics and their benefits for the body. A study conducted among 385 participants from Hong Kong also described the relationship between knowledge about probiotics and specific sociodemographic characteristics, with the higher education level of respondents being positively associated with greater awareness about probiotics [41]. Respondents in the youngest age group (up to 29 years) had the highest degree of knowledge about probiotics and their beneficial effects. It is likely that a larger proportion of parents in this age group (up to 29 years) are under the influence of the National Action Plan on the Decade of Roma Inclusion Initiative 2005–2015, developed on the basis of the strategy for providing a better life for the Roma minority, with a focus on health, education, and employment [42]. Regarding the correlation of the number of children in the family and intake of probiotics, our test showed that the approach of parents to apply probiotics is not influenced by the number of children in individual households. This result confirms that the respondents we studied, regardless of the level of education and economic status, are responsible for the health of each of their children. Despite the limited number of participants, our study once again confirms the connection between education and nutrition culture as part of a healthy lifestyle.

The present study showed that children from Roma families in Bulgaria eat unhealthy food. One of the challenges for minority parents is to build a nutritional culture and habits in their children. The participation of Roma parents in community initiatives related to a healthy lifestyle for children is essential.

In cases where the additional intake of probiotics is necessary, it is advisable for parents to give them to their children. The studied parents can purchase the probiotics from pharmacies as well as from online pharmacies that are registered in accordance with the requirements of the Bulgarian Drug Agency [43].

Nutrition behaviour depends simultaneously on social and economic conditions. The very formation of certain preferences also reflects the structure of society—advertising and public relations [44]. The social aspect is limited to the family impact of the parents on their children. When carrying out the initiatives related to the protection and improvement of health, the socioeconomic aspects must be taken into account, as well as the education of people of Roma origin.

In recent years, the integration of Roma people in education has been one of the state’s priority tasks, and the results are visible among the younger members of the ethnic group.

Roma students studying health specialties based on cultural traditions and acquired academic knowledge can encourage parents and representatives of minorities in general to engaged in proper nutrition and the use of probiotics for the purpose of prevention and treatment. By organizing practical initiatives and seminars, Roma students can contribute to increasing knowledge about probiotics, improving food culture and health education in general among minority groups. In implementing these activities, Roma students face a number of barriers of a cultural, social, and economic nature. The support of institutions will contribute to the more successful implementation of the activities initiated by Roma students.

## 5. Conclusions

The role of Roma students, under the guidance of a mentor and the mediation of a health mediator, is to initiate community actions that will contribute to increasing nutritional and health culture among minority group parents in Bulgaria. Roma students, as participants in the Programme Trust for social alternatives, have improved their communication skills, used their acquired knowledge in practice, and have contributed to raising healthy culture among parents within a minority group.

The activities carried out by the students studying health specialties will be a catalyst for a number of processes beneficial to public health.

The good practices for the implementation of the political framework for the integration of Roma people in the priority areas of education, health care, employment, housing policy, etc., should be accomplished through the cooperation of minority students.

## Figures and Tables

**Table 1:** Null and alternative hypothesis.

	Null Hypothesis	Alternative Hypothesis
1. Hypothesis about differences in the knowledge about what probiotics are and parental age.	There are no differences in the knowledge about probiotics depending on age.	Differences in the knowledge about probiotics are observed between parents in different age groups.
2. Hypothesis about differences in the knowledge about the usefulness of probiotics for health and parental age.	There are no differences in the assessment of the usefulness of probiotics depending on age.	Differences in the assessment of the usefulness of probiotics are observed between parents in different age groups.
3. Hypothesis about differences in knowledge about what probiotics are and parental education.	There are no differences in the knowledge about probiotics depending on education.	Differences in the knowledge about probiotics are observed between parents with different levels of education.
4. Hypothesis about the differences in the knowledge about what probiotics are and the number of children in the family.	There are no differences in the knowledge about probiotics depending on the number of children.	Differences in the knowledge about probiotics are observed between parents with a different number of children in the family.

**Table 2:** Demographic characteristics of the respondents.

Characteristics	Frequency	Percentage (%)
Gender		
Female	73	81.1%
Male	17	18.9%
Education *		
Primary	55	63.2%
Secondary	17	19.5%
Specialized secondary	12	13.8%
Bachelor’s degree	3	3.5%
Children in family		
One child	39	43.3%
Two children	45	50.0%
Three children	6	6.7%
Place of residence		
Parvomay	19	21.1%
Saedinenie	20	22.2%
Perushtitsa	51	56.7%
Type place residence		
City	57	63.3%
Village	33	36.7%

* Percentages are calculated from valid responses only (*n* = 87), excluding 3 missing values.

**Table 3:** School period of the children of the interviewed parents of Roma origin.

	1st Child		2nd Child		3rd Child	
	Frequency	Percentage (%)	Frequency	Percentage (%)	Frequency	Percentage (%)
Elementary school	52	57.8	24	47.1	-	-
Primary school	36	40.0	23	45.1	5	83.3
Secondary school	2	2.2	4	7.8	1	16.7
TOTAL	90	100.0				

**Table 4:** Existence of chronic diseases among the respondents’ children.

	Frequency	Percentage (%)
Stomach diseases	6	6.7
Obesity	8	8.9
Diabetes	2	2.2
High blood pressure	1	1.1
Heart diseases	1	1.1

**Table 5:** Diet of the respondents’ children.

	Never	Once a Week	More than Twice a Week	Every Day
	(%)	(%)	(%)	(%)
Milk and dairy products	1.1	13.3	50.0	35.6
Meat	1.1	11.1	55.6	32.2
Perishable foods	5.6	8.9	45.6	40.0
Fish	31.5	51.7	13.5	3.4
Pasta/Spaghetti	2.2	13.3	46.7	37.8
Food containing hydrogenated fats and sugar	5.6	7.8	33.3	53.3
Fresh fruit	5.6	38.9	26.7	28.9
Vegetables	5.6	31.1	28.9	34.4

**Table 6:** Attitudes toward probiotics.

	Yes	Unsure	No
	(%)	(%)	(%)
They know what probiotics are like	34.4	45.6	20.0
They believe that probiotics are good for your health	34.4	56.7	8.9
Their child has taken probiotics	34.4	29.4	12.2

**Table 7:** Results of hypotheses testing.

Hypothesis	χ^2^	*p*-Value	Result
1	23.309	0	Rejected the null hypothesis
2	9.711	0.008	Rejected the null hypothesis
3	16.186	0.001	Rejected the null hypothesis
4	8.544	0.014	Rejected the null hypothesis

## Data Availability

Data are contained within the article.

## Supplementary Materials

The following supporting information can be downloaded at: https://www.mdpi.com/article/10.3390/healthcare13111314/s1, Supplementary Material S1: Research Questionnaire.

## Abbreviations

The following abbreviations are used in this manuscript.

FAO	Food and Agriculture Organization
REF	Roma Educational Fund
IBD	Inflammatory bowel disease
NEC	Necrotizing enterocolitis
WHO	World Health Organization
