# Supplementary material for: Pilot Study About the Importance of the Active Role of Roma Students: Improving the Health of Bulgarian Children from the Roma Minority Group Through Nutrition and Probiotics
Source: Healthcare (Basel). 2025 Jun 1;13(11):1314. doi: 10.3390/healthcare13111314 (PMC12154747; doi:10.3390/healthcare13111314)
Supplement: Supplementary file 1 [file healthcare-13-01314-s001.zip › healthcare-3577435-supplementary.pdf]

RESEARCH QUESTIONNAIRE

Eating culture among school-age children in the Roma community of Bulgaria and their parents' knowledge about the probiotics

This survey aims to establish the eating habits and nutritional behaviour among school-age children of the Roma community in different regions of Bulgaria.
The questionnaire contains 5 parts:

- Demographic characteristics of the respondents: gender; age; place of residence, number of children to whom the person is a parent, age group of the children
- Presence/absence of chronic diseases in children
- Diet
- Eating regimen and style
- Knowledge about probiotics

The results of the study will be used for scientific purposes and will assess the nutritional behaviour among the Roma community, which will generally contribute to improving the health status and quality of life among adolescents from the Roma community.
The survey is anonymous and voluntary!
By completing the questionnaire, you give informed consent for your data to be processed and used for scientific purposes.

RESPONDENTS' DATA

Location (Please indicate the name of your city / village):
………

Your gender is: □Female □Male
Your age: (Please indicate your age) …………………
Your education (mark with a cross the correct one): □Primary; □Secondary; □Specialized secondary; □Professional-bachelor; □Bachelor; □Higher.
Number of children …………
Your child is in: 1 □ Elementary school; □ Primary school; □ Secondary school.
2 □ Elementary school; □ Primary school; □ Secondary school.
3 □ Elementary school; □ Primary school; □ Secondary school.

PRESENCE/ABSENCE OF CHRONIC DISEASES AMONG CHILDREN

Does your child often suffer from colds?
□ yes
□ no

Does your child have any of the following diseases?

□ diseases of the stomach and esophagus (gastritis, ulcer)

□ obesity, as well as high blood sugar, high blood pressure (metabolic syndrome)

□ diabetes

□ high blood pressure (hypertension)

□ heart disease

DIET

Please mark with a cross (X) what applies to your child

Diet	Never	Once a week	More than twice a week	Every day
How often does your child eat milk and dairy products (cottage cheese, cheese, yellow cheese)?				
How often does your child eat meat?				
How often does your child eat perishable products (sausages, sausages, pate, etc.)?				
How often does your child eat fish?				
How often does your child eat pasta, including macaroni and couscous?				
How often does your child eat products containing hydrogenated fats and sugar (waffles, biscuits, pastry)?				
How often does your child eat fresh fruits (apples, oranges, blueberries, etc.)?				
How often does your child eat vegetables (peppers, carrots, spinach, etc.)?				

What type of fat do you most often cook with?

□ lard

□ sunflower oil

□ margarine

□ olive oil

□ butter

□ coconut oil

EATING REGIMEN AND STYLE

Please mark with a cross (X) what applies to your child

	Yes	Usually yes	Usually no	No
Your child usually eats at home				
Your child eats while watching TV				
Your child eats while playing on their phone/with their toys				
Your child eats on the way to kindergarten/school				
Your child usually eats at kindergarten/school				

KNOWLEDGE ABOUT PROBIOTICS

	Yes	Unsure	No
Do you know what probiotics are?			
Are probiotics good for your health?			
Has your child taken probiotics?			
